# Supplementary material for: Association between oral microbiome and seven types of cancers in East Asian population: a two-sample Mendelian randomization analysis
Source: Front Mol Biosci. 2023 Nov 21;10:1327893. doi: 10.3389/fmolb.2023.1327893 (PMC10702768; doi:10.3389/fmolb.2023.1327893)
Supplement: Supplementary file 1 [file DataSheet1.ZIP › Supplementary Materials/MR plots of saliva/saliva═╝/ovarian cancer/pheno.2484_to_ovarian cancer_forest.pdf]

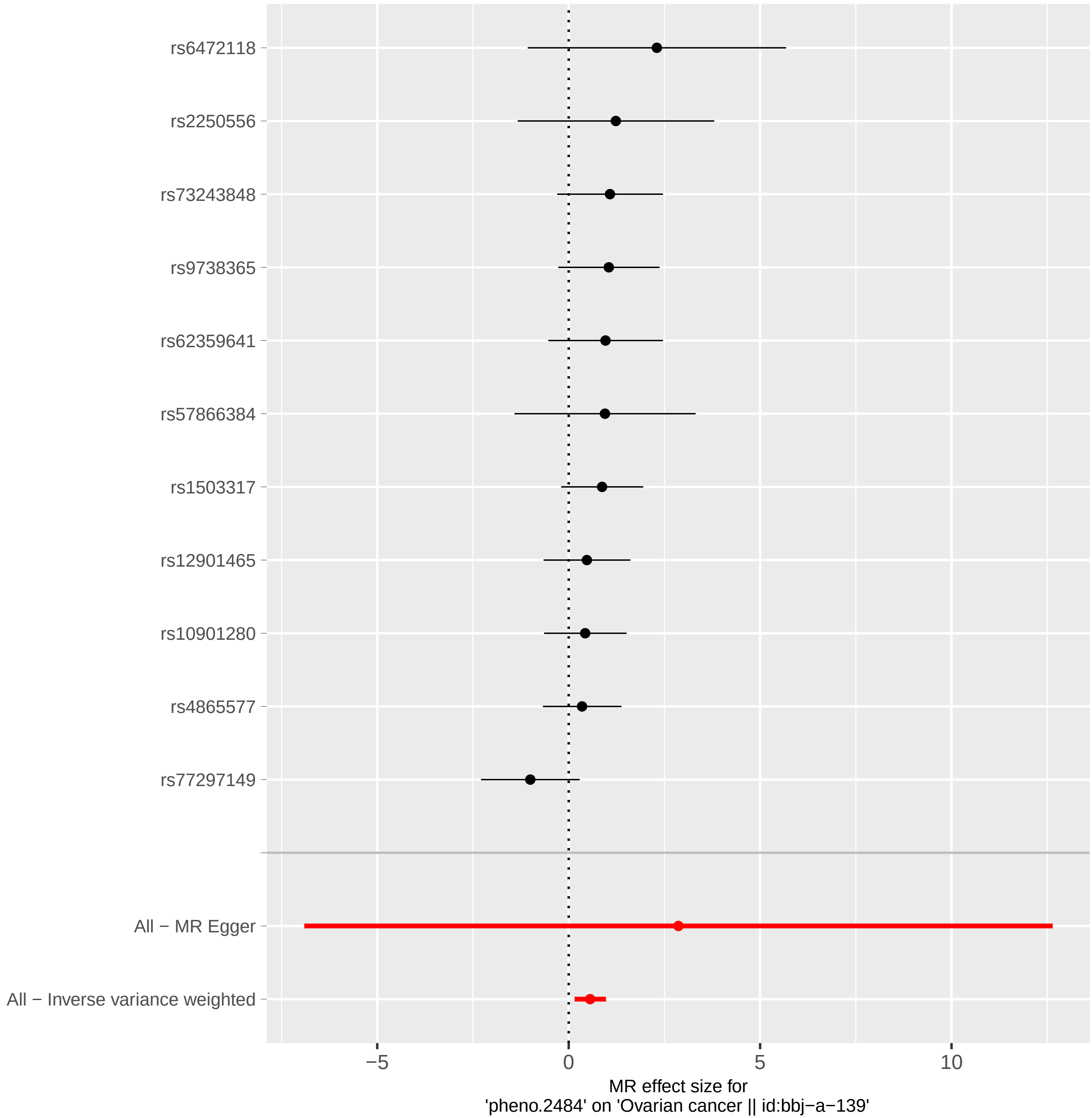
rs6472118
rs2250556
rs73243848
rs9738365
rs62359641
rs57866384
rs1503317
rs12901465
rs10901280
rs4865577
rs77297149
All − MR Egger
All − Inverse variance weighted
−5
0
5
10
MR effect size for
'pheno.2484' on 'Ovarian cancer || id:bbj−a−139'
